# Spinal muscular atrophy disease: a literature review for therapeutic strategies

**Published:** 2010-02-25

**Authors:** M Stavarachi, P Apostol, M Toma, D Cimponeriu, L Gavrila

**Affiliations:** University of Bucharest, Institute of Genetics, BucharestRomania

**Keywords:** neuromuscular disorder, SMN gene, therapeutically drugs, mutations

## Abstract

Currently, there is no cure for the treatment of spinal muscular atrophy (SMA). Based on the available clinical and molecular findings, different therapeutic strategies were tested *in vitro* and *in vivo* and clinical trials are ongoing. The main therapeutic direction is focused on the enhancement of *SMN* expression by increasing the full–length (fl) SMN2 transcript levels, preventing the SMN exon 7 from skipping or from protein stabilizing. In addition, the action of neurotrophic, neuroprotective or anabolic agents is tested and stem cell and gene therapy approaches are in a promising development.

## Introduction

Spinal muscular atrophy is the second most common lethal autosomal recessive disorder in Caucasians, after cystic fibrosis [1 , 2]. The disease is characterized by the progressive degeneration of the alpha motoneurons, which leads to muscle atrophy, paralysis and even death [3]. Several clinical types were described for SMA, according to the age onset and the progression of the symptoms. The most recent classification [4] includes four types: type Ⅰ (Werdning–Hoffman), type Ⅱ (intermediate), type Ⅲ (Kugeleberg–Welander) and type Ⅳ (adult form). Type Ⅰ is the most severe form, with an onset before the age of 6 months and a life expectation of around 2 years. Types Ⅱ and Ⅲ are known as chronic forms and are less severe, with an onset between 6–18 months (type Ⅱ) and respectively after 18 months (for type Ⅲ). In many cases, Type Ⅳ mimics the symptoms of type Ⅲ, but the onset is typically around 30 years old. A normal life expectancy is characteristic for this adult form. Some research studies also reported the existence of type 0 or embryonic form, which is characterized by reduced movement of the fetus between 30–36 weeks of the pregnancy and a very short life expectancy [5].

The SMA determining gene, termed survival motor neuron (*SMN1*) was mapped on 5q11.2-13.3 region [6]. The homozygous deletion of *SMN1* exon 7 is the most common mutation found in SMA patients, but there are also many cases of compound heterozygous subjects, for whom deletions and different point mutations have been detected. The *SMN1* paralog and *SMN2 *, differ with a single nucleotide in exon 7 (C–T), that alters the splicing process. A few models were proposed in order to explain this mechanism. The first hypothesis sustains the existence of an Exonic Splicing Enhancer (ESE) in *SMN1* gene, known as a binding site for splicing regulatory factors like arginine/serine rich (SR) protein. In *SMN2* gene, the T nucleotide in position 6 of exon 7 disrupts this ESE and, in an implicit way, the binding of the SR protein [7 ,8 , 9 ,10]. Another model proposed the existence of an Exonic Splicing Silencer only in *SMN2* gene, which is a binding site for the hnRNP A1 repressor protein [ 11]. An extended analysis of these two models confirm the first one and sustain the role of hnRNP A1 protein in exon 7 skipping, but for both genes, not only for the SMN2. A new hypothesis emerged from these observations sustains that the hnRNP A1 protein acts in a general manner to block splicing of exon 7, in the absence of SF2/ASF dependent ESE [12 ]. No matter which of these models is the real one, the result of the splicing factors action was obvious: only 20% of the SMN2 gene transcripts contain a message for the fl–SMN protein, while the remaining 80% are responsible for a truncated form. In SMA patients, the fl–SMN protein is synthesized in a small quantity, while the truncated protein is degraded and unable to sustain the motor neuron survival.

These molecular mechanisms indicated that *SMN2* gene could be a good target for SMA disease therapy, meaning the increase of fl–SMN protein level. Different compounds are tested in order to accomplish this aim. However, alternative strategies using neuroprotective agents, stem cells transplantation or gene therapy are ongoing.

## Enhancement of SMN expression

The ability of SMN2 gene to encode for the fl–SMN protein has been hardly exploited, by several mechanisms:

- Increasing the fl-SMN transcript level by activating SMN2 gene promoter
  Some researchers reported that the promoters of the SMN genes have similar positive regulatory elements [13]. This information was extremely useful in finding the compounds able to increase the fl–SMN2 transcript level and, in an implicit way, the normal fl–SMN protein quantity. It is well known that histone acetyltransferases (HATs) enhance the transcription through the chromatin relaxation, while the histonedeacetylases (HDACs) induce the transcriptional repression by chromatin condensation [ 14]. In this context, HDAC inhibitors became good candidates in finding a way to increase the fl–SMN level. For the first time, these kinds of compounds were used in 2001: a revolutionary increase of fl–SMN2 transcript level could be noticed following the sodium butyrate treatment of SMA patients, derived cell lines and SMA knockout transgenic mice [15]. Additional researches indicated that the half–life of sodium butyrate does not recommend its use in clinical trials. Anyway, these studies concerning the effect of sodium butyrate on SMN gene expression were the beginning for new researches in testing the action of HDAC inhibitors compounds on SMN expression.
  A sodium butyrate derivate (sodium 4 phenylbutyrate–PB) has been tested on fibroblast cells derived from SMA patients [16] and then in clinical trials [ 17,18 ]. The emerging conclusion was that PB improves motor neuron function in SMA type Ⅱ patients, by enhancing the fl–SMN transcript and protein levels. Although patients with urea cycle disorders [19] tolerate well this compound, its clinical use in SMA is still reserved, because of its short half–life of approximately 1 hour.
  Another HDAC inhibitory compound with a promising effect in SMA treatment is hidroxyurea. In cultured lymphocytes from SMA patients, hidroxyurea is able to increase the ratio of full–length to truncated SMN messenger RNA, the SMN protein level and intranuclear gems number (the structures containing SMN protein) [20]. Based on this data, clinical trials were opened in United States [21 ] and Taiwan [22]. Preliminary results showed an increase of fl–lSMN mRNA level at only 8 weeks of treatment.
  The most studied HDAC inhibitor in SMA treatment is valproic acid (VPA) also known as 2 – propylpentanoic acid. This drug was already used in the treatment of epilepsy and with therapeutically indication in bipolar disorders. Taking into account that VPA is a fatty acid and a HDAC inhibitor, just like sodium butyrate, it was hypothesized that it may increase SMN2 mRNA level. If sodium butyrate has a short half–life of only six minutes in human serum, the VPA has the advantage of 8 to 10 hours half–life. The administration of VPA in therapeutic doses (0.5–1000ॖM), on fibroblast lines in SMA patients, for a 16–hour period, has determined the increase of fl– SMN2 mRNA level from 2 to 4 fold. As it was expected, a stronger response was obtained when VPA low doses (0.5–5॥M) were administrated on fibroblast lines in SMA patients with more than two SMN2 copies. By testing the action of this drug on rat organotypic hippocampal slice cultures (often used for Central Nervous System disorders studies), it was proved that VPA also increases SMN level in the neuronal tissue [23,24]. It is presupposed that VPA action is mediated by binding AP1 and SP1 transcription factors through the human and rat SMN promoter [23]. The VPA effects on neurogenesis and astrocyte proliferation, as well as on increased levels of Bcl–2 and Bcl–x anti–apoptotic factors, were also discussed in researches involving type Ⅲ like–SMA mouse models [25]. These in vitro and in vivo studies have encouraged the opening of VPA clinical trials. Consequently to the pilot trial which showed an increased SMN protein level in healthy carriers as well in SMA patients [26], four clinical trials with VPA and carnitine (to avoid the possible toxicity of the drug) have been started [27].
  HDAC inhibitors from the second generation like Trichostatin A, SAHA, M344 benzamide, MS–275, m–Carboxycinnamic acid, bis–lHydroxamide were included in several recent studies [28 , 29]. Of these compounds, SAHA seems to be the most promising drug for therapy, as it increases SMN levels at low concentrations, has a favorable profile citotoxicity and it is well tolerated [29].
- Preventing the SMN2 exon 7 from skipping
  Researches regarding the molecular mechanism of SMA physiopathology revealed that the production of the SMN truncated protein is the result of an alternative splicing of exon 7 in the majority of patients. Preventing this exon from skipping is one of the most common ways to increase the full–length of SMN protein level.
  The first identified compound that helps in preventing splicing of the endogenous SMN2 exon 7 is sodium vanadate, an inhibitor of protein tyrosine phosphatases, alkalin phosphatases and ATPases. Among several signaling pathways modulators on splicing, only treatment with sodium vanadate (50–100microM) for 10 to 24 hours showed an important increase in fl–SMN2 mRNA. The treatment with 50microM sodium vanadate caused the highest amount of the fl–transcript, while a concentration over 100microM resulted in the cell's death. The long treatment period is explained by the time needed for cellular processes like transcription or phosphorylation. In this context, it is hypothesized that the phosphorylation of some cellular factors may be involved in SMN2 splicing process and that the most probable proteins influenced by sodium vanadate treatment are SR, snRNP and hnRNP [30].
  The HDAC inhibitors have effects not only on chromatin relaxation, but also on SMN exon 7 retaining, as it could be noticed in the case of sodium butyrate treatment on lymphoid cell lines. According to this treatment, the SR protein induction was detected [15]. This dual action exerted on SMN protein level was also observed in the case of VPA and Trichostatin A.
  Aclarubicin, a drug from anthracylcine antibiotic class is frequently used in chemotherapy against solid tumors and leukemia. However, aclarubicin seems to significantly increase the gems number and the SMN level in a SMA Ⅰ patient derived fibroblasts, by altering the splicing process. The high toxicity profile has embedded the use of this drug in treating SMA patients [31 ].
  Trying to avoid the discovery of the potential therapeutic drugs that cannot cross the blood brain barrier, a recent research [ 32] has focused on the action of some polyphenol botanical compounds with known ability in passing this difficulty. Curcumin, resveratrol and epigallocatechin galate were previously reported to have some modifying actions in neurodegenerative diseases [33]. An increase in fl–SMN transcripts and protein levels was noticed after the administration of these compounds in cultures of SMA derived fibroblast cells. Additionally, it seems that curcumin up–regulate some genes encoding for SR proteins, essentially in splicing process.
  Among natural or synthesized compounds, physical exercises are reported to be involved in the SMN2 splicing regulation, probably by modifying the expression pattern of pre– mRNA splicing factors in motoneurons [34]. Another study based on type 2 SMA mouse model proved that physical exercises have benefic effects in motor neuron protection, acceleration of muscle maturation and lifespan gain through the NMDA–receptor activity modulation [35]. A combined therapy of drugs and regular physical exercises is thought to improve clinical signs in SMA patients.
- Stabilizing SMN Protein
  In order to identify the compounds able to stabilize the SMN protein, by acting at the translational level, a high throughput screening of approximately 47000 compounds with potential effect on SMN protein was performed. Only indoprofen increases the SMN protein level in the treated fibroblast cells with about 13 percent, reported to untreated cells [36]. Moreover, as a result of indoprofen treatment, the number of gems was enhanced in vitro and a reduced embryonic lethality was noticed in SMA mutant mice.
  SMN stabilizing protein compounds are also aminoglycosides. This class of antibiotics have in vitro capacities to suppress premature stop mutations in some genes (e.g. CFTR [37 ] and dystrophin genes [38]) and to affect the translation termination process, by altering the conformation of ribosomal decoding site [39]. Based on these findings, a research group reported an important increase of SMN protein level of fibroblast treatment with tobramycin and amikacin [40]. Other six aminoglycosides which were found to increase SMN protein level in fibroblast cells [41] may be used for therapeutic purposes.
  There are also studies indicating that proteasome inhibitors may have a benefic effect on SMN protein stability [42]. It is already known that the ubiquitin/proteasome pathway has an important role in the proteolysis of intracellular proteins. By using pulse–chase analysis, it was confirmed that SMN protein is ubiquitinated and degraded by the ubiquitin/proteasome system [43].

## Neurotrophic, neuroprotective and anabolic agents' administration

Neurotrophic factors have been referred to be good candidates for therapeutic strategies of several motor neurons diseases, based on the ability to slow down motor neuron death and axon degeneration [44]. Cardiotrophin–1 is a cytokine from interleukin–6 family, with known benefic effects on survival of motor neurons during embryonic period [45]. The cardiotrophin–1 delivery to SMA mutant mice revealed that the protective role for proximal and distal motoneurons is also present in postnatal period [46].

Neuroprotective agents were proposed for clinical trials in SMA, following the in vitro and in vivo studies. Because a hypothesis regarding SMA pathogenesis includes glutamate citotoxicity, factors with anti-glutamate action have been tested in order to improve the motor skills and life expectancy. Gabapentin is an excitatory amino acid neurotransmitter with neuroprotective properties, proposed to have therapeutic effects for Amyotrophic Lateral Sclerosis (ALS). Two clinical trials with gabapentin were developed for patients with SMA types Ⅱ and Ⅲ, for a one–year period. If the first gabapentin study (developed on 84 SMA patients) has not revealed any potential therapeutic effects [47], the second one (applied on 120 patients) revealed a small improvement of muscle strength [48].

Another anti–glutamate factor is riluzole, a compound with neuroprotective effects tested in a SMA mutant mouse model [49]. The first clinical trial of riluzole in SMA patients was started in 2003 [50] and enrolled 10 SMA type Ⅰ patients. The authors of this study concluded that it is safe to use riluzole in infants, but a more statistical powerful study is needed to ascertain the benefactor effects of this drug. At this moment, two riluzole trials are ongoing on SMA type I patients in USA and in France [51].

Anabolic agents such as albuterol (also known as salbutamol) were reported to have significant results in clinical state improvement. Myometry, forced vital capacity and lean body mass were increased after 6–months of oral administration [52]. Starting from these clinical findings, a few years later, another research group studied these effects of the drug and reported an increase of SMN mRNA and protein level in fibroblast cells derived from SMA patients [53]. It is thought that the salbutamol is acting on SMN protein through the influence of the SMN2 exon 7 splicing event.

It was recently reported that Follistatin [54] is an anabolic agent with an ability to increase muscle mass, to improve motor neurons' function and to prolong life survival, in SMA mutant mice. Taking into account that the SMN protein level was not increased by follistatin administration, it was suggested that this glycoprotein exerts its beneficial effects by a SMN independent mechanism. It is thought that follistatin is acting, at least in part, through a myostatin (a TGF–B family member) pathway, a negative regulator of muscle mass. The over expression of follistatin in myostatin null mice, results also in an enhancement of muscle mass, suggesting the existence of additional pathways or acting mechanisms [55].

A combined therapy of neurotrophic, neuroprotective or anabolic agents may lead to the improvement of SMA phenotype and, in an implicit manner, to the lifespan duration.

### Stem cell transplantation and gene therapy

Stem cell transplantation and gene therapy represent a promising future in the treatment of motor neuron diseases, including SMA. It is known that neuronal stem cell transplantation can lead to an improvement of motor neuron disease phenotype, through different mechanisms, such as replacement of non–neuronal cells [56], delivery of neuroprotective factors [57] or reduction of toxic compounds [58]. For the first time, stem cells transplantation was used in a SMA model as putative therapeutic pathway in 2008 [59]. The motor neuron function improvement and survival of SMA mice led to the conclusion that neural stem cells transplantation has positive effects on SMA disease phenotype.

Lesbordes was reported to have conducted the first gene therapy study on SMA in 2003, [46]. He used the intra–muscular injection with an adenoviral vector expressing cardiotrophin–1, whose effects were discussed previously in this paper. The method had been tested few years earlier [60], when cardiotrophin–1 appeared to be a protective factor in progressive motor neuronopathy model mice. In 2004, a new gene therapy study was reported: a lentivector developed from infectious equine anemia virus was used in gene transfer in SMA mice, consequently in *in vitro* testing. The SMN lentivector was injected in two days of age SMA mice at the level of the voluntary muscles. Following this therapy, the SMA mice life expectancy was increased with 3 to 5 days and a motor neuron death reduction could be recorded [61].

Remarkable results for SMA potential therapy were obtained by using RNA strategies. The enhancing of exon 7 incorporation in SMN message was found to be a successful strategy in the increase of SMN protein level, but drugs such as sodium vanadate, sodium butyrate and aclarubicin were not recommended because of their short half–life or toxicity. A bifunctional antisense oligonucleotides strategy was developed in order to increase SMN2 gene expression [62]. The antisense oligonucleotides (about 20 nucleotides length) are complementary to SMN2 exon 7 sequence but also contain non–complementary tails, able to bind splicing factors and to stimulate the natural splicing reaction. Applying this strategy, additional to the action on SMN2 exon 7 splicing, a partial restoration of gems number in SMA fibroblast cells could be observed. In vivo, delivery of bifunctional RNAs was possible by using plasmid–based and rAAV recombinant adeno–associated virus vectors [63]. The advantage of rAAV vectors is the high tropism for myocites and neuronal cells and the retrograde transport to neurons after the intramuscular injection. An improvement of the antisense oligonucleotides strategy was the use of modified U7snRNA, as vehicle for SMN antisense, RNA complementary to the 3'splice site of SMN exon 8 [64]. This way, the researchers were able to identify anti SMN U7snRNAs that can alter the splicing process and stimulate the SMN2 exon 7 retaining. Compared to the others antisense oligonucleotides strategies that use chemical modified RNAs, the SMN U7snRNAs provide the advantage of the continuous delivery in the cell and the increased half–life of the RNAs.

Another RNA strategy for preventing SMN2 exon7 from skipping is known as trans – splicing system. Trans–splicing is a natural but rare process that has been recently induced in therapeutic assays for cystic fibrosis [65] or Alzheimer disease [66]. This strategy is based on the competition for splices sites between endogenous RNA (mutant) and the therapeutic RNA (able to provide the correct RNA sequence through a trans splicing process). Initially, plasmids and rAAVs were used for delivery of trans splicing RNAs, in order to restore small nuclear ribonucleoprotein (snRNP) assembly in vitro [67]. As this method had no results *in vivo*, a new system was developed: a single vector that co–expresses the trans splicing RNA. This way, antisense RNA is able to increase the snRNP assembly and SMN level protein in vitro as well as *in vivo* [68]. Although the regulatory networks for this constructs are not yet clarified, the results are promising and there is a hope of improving therapeutic strategies for SMA, life expectancy and quality of life for patients.

## Conclusions

A large spectrum of compounds that act through various mechanisms were tested in order to identify a therapeutically drug in treating spinal muscular atrophy disease. However, no compound is currently available in SMA therapy, as toxicity and half–life duration are some of the limiting factors. A special care is needed when results obtained in animal models are extrapolated in clinical trials on humans, taking into account the genetics and physiopathological differences between species. The stem cell and gene therapy give promising results, but the confirmation for a drug and its way to delivery, are still being waited for.
